# Detection of SARS-CoV-2 Antigens in the AV-Node of a Cardiac Conduction System—A Case Report

**DOI:** 10.3390/tropicalmed7030043

**Published:** 2022-03-04

**Authors:** Hrvoje Jakovac, Antun Ferenčić, Christophe Stemberger, Bojana Mohar Vitezić, Dražen Cuculić

**Affiliations:** 1 Department of Physiology, Immunology and Pathophysiology, Faculty of Medicine, University of Rijeka, 51000 Rijeka, Croatia; 2 Department of Forensic Medicine and Criminalistics, Faculty of Medicine, University of Rijeka, 51000 Rijeka, Croatia; antun.ferencic@medri.uniri.hr (A.F.); drazen.cuculic@medri.uniri.hr (D.C.); 3 Department of Pathology, Clinical Hospital Centre Rijeka, 51000 Rijeka, Croatia; chstemberger@medri.uniri.hr; 4 Department of Microbiology and Parasitology, Faculty of Medicine, University of Rijeka, 51000 Rijeka, Croatia; bojana.mohar@medri.uniri.hr

**Keywords:** COVID-19, SARS-CoV-2, spike protein, nucleocapsid protein, AV-node, cardiac conduction system, cardiac arrhythmia

## Abstract

Mounting evidence indicates that new arrhythmic events frequently occur during and after coronavirus disease (COVID-19), posing additional mortality risk in older-aged and critically ill patients. However, the underlying mechanisms and cardio pathological substrates of COVID-related arrhythmias have not been clarified yet. Here, we report findings of severe acute respiratory syndrome coronavirus 2 (SARS-CoV-2) antigens and genes in the atrioventricular node (AV-node) of a cardiac conduction system, pointing to its direct infection as a possible arrhythmogenic factor.

## 1. Introduction

Severe acute respiratory syndrome coronavirus 2 (SARS-CoV-2) is a highly pleiotropic virus that causes coronavirus disease (COVID-19), a systemic disease most strikingly affecting the respiratory system and blood vessels; however, the function of almost all body tissues can be impaired in infected persons. The fatal outcomes most often result from acute respiratory distress syndrome (ARDS) and associated cytokine storm-driven systemic inflammatory response syndrome (SIRS), leading to the multiorgan failure syndrome (MOFS). Furthermore, not uncommonly, the sudden development of thrombotic/thromboembolic events also can be the causative factor of lethality in COVID-19 patients. Major factors increasing the risk of a fatal outcome are older age and the presence of comorbidities such as obesity, diabetes mellitus, and arterial hypertension. These conditions are considered to prefer a hyperinflammatory response after initial viral immunoevasion, rendering a milieu suitable for the development of an auto-aggressive cytokine storm. Underlying mechanisms of inflammatory facilitation in such cases are thought to include loss of insulin anti-inflammatory and immunomodulatory signaling in diabetic patients, excessive release of pro-inflammatory adipokines in obese patients, and impairment of renin-angiotensin system (RAS) with proinflammatory angiotensin II (Ang II) prevailing over anti-inflammatory angiotensin 1–7 (Ang1–7) in hypertensive patients. On the other hand, all of these conditions, especially if untreated or treated improperly, have been recognized as major risk factors for cardiovascular and, consequently, pulmonary function impairment, leading eventually to the discrepancy between actual tissue oxygen needs and its delivery. In states of acutely impaired blood oxygenation, as occurring during COVID-19, such settings may also contribute to a higher likelihood of a poorer outcome.

Lately, distinct types of new-onset cardiac arrhythmias have emerged as a common finding in COVID-19 patients, being concerning especially in critically ill patients as a possible additional factor worsening the outcome [1,2]. The most frequently reported arrhythmias among COVID-19 patients are tachyarrhythmias, atrial fibrillation/flutter, and bradyarrhythmias with different degrees of atrioventricular block [1,2,3]. Despite the fact that SARS-CoV-2 has been found in the working myocardium of infected subjects, COVID-19-related arrhythmias are thought to be elicited secondary to severe systemic illness, cytokine storm, and hypoxic conditions, and are not as a consequence of direct viral infection [2]. Still, it has recently been reported that complete atrioventricular block can emerge in COVID-19 patients not presenting with the severe inflammatory response [3]. Here, we present the immunohistochemical detection of SARS-CoV-2 spike and nucleocapsid antigens, as well as the finding of viral genes in the cardiac atrioventricular node (AV-node) of a patient who died from COVID-19 and had an abnormal heart rhythm prior to death, pointing to the possibility of direct viral infection of the cardiac conduction system components as being a potential arrhythmogenic mechanism of COVID-19-elicited arrhythmias.

## 2. Case Description

An 85-year-old diabetic Caucasian man that was unconscious was admitted to the emergency room at Clinical Hospital Center Rijeka (CHCR, Rijeka, Croatia) after he had been found in his apartment. While being transported, he received high-flow oxygen therapy (15 L/min) and was given 500 mL of saline intravenously. Upon admission, the patient appeared to be in critical condition, achieving a score of 5 on the Glasgow Coma Scale (GCS). A rapid SARS-CoV-2 antigen test of a nasopharyngeal swab showed a positive result, further confirmed by RT-PCR testing. Physical examination revealed tachypnoea (50/min), tachycardia (160/min), severe hypotension (50/30 mmHg), diffuse skin marmorization with acrocyanosis, and prolonged capillary refill (>4 s). Auscultatory, intensity of breath sound was considerably weakened above the entire lungs. Oxygen hemoglobin saturation was indeterminable using a pulse oximeter, but arterial blood gas analysis found pO_2_ of 4.2 kPa, sO_2_ of 51.7%, pCO_2_ of 4.1 kPa, and pH of 7.30. Other laboratory tests were within normal ranges, except elevated CRP (60 mg/L), low leukocyte count (3 × 10^9^/L), and high urea (BUN; 17.9 mmol/L) and creatinine (204 μmol/L). His plasma glucose concentration was 5.2 mmol/L. He was reportedly being treated with insulin, but data on treatment duration and dosing regimen, as well as anti-SARS-CoV-2 vaccination status, were not available. Chest X-rays revealed bilateral, diffuse, reticular opacities infiltrating the lung tissue. An electrocardiogram showed atrial tachycardia, left posterior fascicular block (LPFB), and right bundle branch block (RBBB).

**Figure 1:**
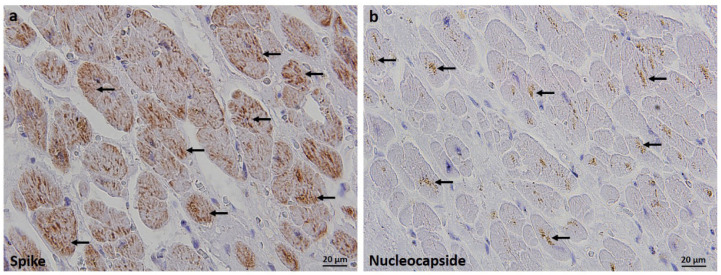
Immunohistochemical detection of severe acute respiratory syndrome coronavirus 2 (SARS-CoV-2) spike protein (**a**) and nucleocapsid protein (**b**) in the cardiac atrioventricular node (AV-node) obtained from a patient who died from COVID-19 and had an abnormal heart rhythm prior to death. Spike protein expression was diffusely present in the cytoplasm of conducting cardiomyocytes, resembling endoplasmic reticulum arrangement and rendering a “tigroid” appearance, (**a**) shown with arrows. Nucleocapsid immunopositivity was less abundant and showed scattered punctiform staining patterns, frequently forming juxtanuclear aggregates, shown with (**b**) arrows. Staining was performed using the rabbit anti-SARS-CoV-2 spike glycoprotein antibody (Abcam, Cambridge, UK, ab272504; dilution 1:4000) and mouse anti-SARS-CoV-2 nucleocapsid protein antibody (Cell Signaling Technology, Danvers, MA, USA, clone E8R1L; dilution 1:200). Immunoreactions were visualized by DAKO EnVision+System (DAKO Cytomation, Santa Clara, CA, USA). Magnification × 600. Tissue was obtained from the base of the atrial septum in the Koch triangle. Prior to death, the patient had atrial tachycardia, left posterior fascicular block (LPFB), and right bundle branch block (RBBB).

The patient immediately underwent rapid sequence intubation (RSI) and volume-controlled continuous mandatory ventilation (V-CMV). He was extensively rehydrated (plasmalyte 1000 mL intravenously) and hemodynamically supported by noradrenaline (5 mg/50 mL). Despite the best available approach, the patient’s condition was rapidly deteriorating and he died due to cardiopulmonary arrest 4 h after admission. Autopsy ascertained acute respiratory distress syndrome with lung tissue positivity for SARS-CoV-2, proven by RT-PCR and immunohistochemistry. Therewithal, subendocardial spindle-shaped tissue sampled from the paraseptal area of the right atrium within the apical part of the Koch triangle exhibited notable SARS-CoV-2 spike protein and nucleocapsid protein immunohistochemical positivity in the bundles of conducting cardiomyocytes (Figure 1).

Spike protein staining signals were widely intertwined throughout the cytoplasm, resembling endoplasmic reticulum arrangement and rendering a “tigroid” appearance (Figure 1a, arrows). SARS-CoV-2 nucleocapsid immunopositivity was considerably less abundant and showed scattered punctiform staining patterns, frequently forming spotty juxtanuclear aggregates (Figure 1b, arrows). Tissue staining was performed using rabbit anti-SARS-CoV-2 spike glycoprotein antibody (Abcam, UK, ab272504; dilution 1:4000) and mouse anti-SARS-CoV-2 nucleocapsid protein antibody (Cell Signaling Technology, USA, clone E8R1L; dilution 1:200). Visualization of immunoreactions was rendered by DAKO EnVision+System (DAKO Cytomation, USA). In addition, using AccuPower^®^ SARS-CoV-2 Multiplex Real-Time RT-PCR Kit (Bioneer, Daejeon, Korea), SARS-CoV-2 genes were detected in the specimen of the equally localized cardiac conduction tissue at relatively low cycle threshold (Ct) values (E gene Ct = 30.01; RdRp/N gene Ct = 28.59). There was no inflammatory infiltration throughout the cardiac tissue, but mild interstitial edema was found. Coronary arteries showed a moderate degree of atherosclerotic changes, consistent with age and diabetes, with no thrombotic occlusions, capillary dilations, and microhemorrhages being present.

## 3. Discussion

Despite the increasing number of epidemiological and clinical studies reporting a link between COVID-19 and new-onset cardiac arrhythmias, the mechanism of their development during SARS-CoV-2 infection is still obscure. A recent meta-analysis showed that pooled incidence of cardiac arrhythmias is 16% among overall COVID-19 patients, being the second (after myocardial infarction) most common cardiac complication of COVID-19 [4]. The proposed pathophysiological mechanism involved in the development of the COVID-19-related arrhythmia includes hypoxemia, myocardial ischemia/hypoxia, systemic inflammation with hypercytokinemia (cytokine storm), dyselectrolytemias, and side effects of some antiviral drugs [5,6]. As COVID-19 is a systemic hyperinflammatory disease significantly impairing respiratory function, and the latter factors have long been known as being pro-arrhythmic in many other clinical entities, these assumptions are fully justified, while the mentioned mechanisms most likely contribute to the development of COVID-19-driven arrhythmias. However, mechanistic studies on the arrhythmia pathogenesis in the specific context of SARS-CoV-2 infection are lacking, and an abnormal heart rhythm has been found to be frequent in recovered patients [7], as well as in patients with mild symptomatology [3], who are not hypoxemic and do not have a hyperinflammatory response.

Here, to the best of our knowledge, we, for the first time, reported detection of SARS-CoV-2 antigens and genes in a particular component of the cardiac conduction system, which suggests its infection and viral-mediated dysfunction as a possible cause of COVID-19-related arrhythmias. However, the contribution of other known factors exerting secondary arrhythmogenic effects cannot be excluded, peculiarly in critically ill patients, such as the one described here. Previous autopsy studies detected the SARS-CoV-2 genome in the cardiac tissue of approximately 60% of patients who died from COVID-19 [8,9], with myocardial RT-PCR Ct values correlating positively with pulmonary viral load [8]. However, viral antigens were predominantly localized in interstitial cells of a probable monocyte origin, but only occasionally in cardiomyocytes [8,9], which raised the question of whether cardiac RT-PCR positivity occurred due to parenchymal viral load, or if it was caused by virions from the residual blood [8]. Based on this case, we cannot speculate about the prevalence of cardiac conduction muscle infection among overall SARS-CoV-2 infected persons. Further studies on this topic would be useful to address these issues.

## 4. Conclusions

SARS-CoV-2 can infect cardiac conducting cardiomyocytes from the AV-node. This finding points to the direct infection of cardiac conduction tissue as being a possible arrhythmogenic stimulus in COVID-19 patients. 

## Data Availability

The data are not publicly available due to the protection of patient privacy and adherence to ethical principles. Clinical and laboratory data may be provided upon reasonable request to the corresponding author.
